# Mr. Custance, on Extracting Teeth

**Published:** 1801-09

**Authors:** Geo. Custance

**Affiliations:** Kidderminster


### *To the Editors of the Medical and Physical Journal.*

Gentlemen,

PERMIT me, through the channel of your valuable Journal, to request a *drawing*, or more minute description, of an instrument for extracting Teeth, invented by Mr. Whitford, and strongly "recommended to the attention of the Medical world," by Mr. Reece, of Craven-street. Being much in the practice of extracting Teeth, I have always considered it a desideratum to meet with an instrument which would take out a tooth, not loosened by disease, with less pain than those in common use.—Many such have been proposed by ingenious men, which have equally failed in the practical application of them. The first invention for raising a tooth perpendicularly, I believe, was published by the late Dr. John Aitken, of Edinburgh, in his "Essays on Important Subjects in Surgery;" but neither this, nor any other similar instrument, has been found to answer the purpose, till the one now invented by Mr. Whitford, which Mr. Reece assures us he has "repeatedly made use of with great satisfaction to himself," and that "his patients have uniformly expressed their surprise at the slight pain they experienced in the operation." I confess Mr. R's great success very much *surprises* me, if the *peculiarity* of this instrument consists, as Mr. R. describes, in "the heel of the instrument being applied to the tooth, exactly opposite the point of the claw." This may be done in the common key instrument, but with the almost certain effect of breaking off the corona of a firm tooth. For it is obvious, that in such an application of the instrument the resistance on one side of the tooth is equal to the force applied to the other; and, consequently, that nineteen teeth out of twenty, if their fangs are firm in the alveolar processes, will inevitably be broken. But by placing the fulcrum or heel of the key instrument considerably lower than the fixed point of the claw, the tooth is made to turn on its axis, and may

 generally generally be easily extracted, though the alveoli must be broken. The merit of Mr. Whitford's instrument must then, I humbly conceive, depend upon the peculiar manner in which the action of the fulcrum and lever is performed, and which Mr. Reece will, perhaps, in a future number of your Journal, obligingly describe. To me it appears impossible to *apply* any instrument to the farthest molards, which can raise them in a perpendicular direction; nor can I conceive how any tooth can be thus extracted without the fulcrum resting on a neighbouring tooth, to the manifest risque of injuring it. The theory of raising teeth perpendicularly is pretty, but, I apprehend, can never be reduced to practice by any instrument, excepting when the teeth are loose; for, when they stand firm, such force must be applied to raise them perpendicularly as would greatly endanger whatever the fulcrum rested upon. When a tooth (as often happens) is entirely broken down to the gum on one side, and a bare shell is left on the other, the placing the claw and heel of the instrument opposite to each other would immediately snap it off, without stirring the fangs, provided they stood firm.—That loose stumps and loose whole teeth may be extracted in nearly a perpendicular line, I can easily conceive; it is often practicable with a pair of forceps. The difficulty and objections to attempts of this sort apply principally to the extracting teeth which stand firm in their sockets, or are entirely broken away on one side, and more especially when they are level on both sides with the gum, which frequently happens. I should be happy to be informed by Mr. Reece whether he has found the instrument to answer under all these circumstances, and request he will have the goodness to give the public a more particular description of its mode of action in "elevating the tooth in nearly a perpendicular direction."

I am, &c.

GEO. CUSTANCE.

*Kidderminster, Aug.* 14, 1801.

CRITICAL

